# Association of Trimethylamine N-Oxide Levels and Calcification in Culprit Lesion Segments in Patients With ST-Segment–Elevation Myocardial Infarction Evaluated by Optical Coherence Tomography

**DOI:** 10.3389/fcvm.2021.628471

**Published:** 2021-02-24

**Authors:** Jiannan Li, Yu Tan, Peng Zhou, Chen Liu, Hanjun Zhao, Li Song, Jinying Zhou, Runzhen Chen, Ying Wang, Xiaoxiao Zhao, Yi Chen, Hongbing Yan

**Affiliations:** ^1^Department of Cardiology, Fuwai Hospital, National Center for Cardiovascular Diseases, Peking Union Medical College, Chinese Academy of Medical Sciences, Beijing, China; ^2^Xiamen Cardiovascular Hospital, Xiamen University, Fujian, China; ^3^Fuwai Hospital, Chinese Academy of Medical Sciences, Shenzhen, China

**Keywords:** trimethylamine N-oxide, calcified lesion, optical coherence tomography, ST- segment elevation myocardial infarction, biomarker

## Abstract

The presence of calcified plaques is one of the pathological phenotypes of acute coronary syndrome (ACS) and can be frequently found in culprit lesion segments. Trimethylamine N-oxide (TMAO) is reported to be involved in vascular calcification and plaque instability. This study investigated the relationship between plasma TMAO levels and calcified lesions in culprit lesion segments in STEMI patients. A prospective series of 179 patients with STEMI were enrolled, and calcified lesions from 127 patients were analyzed by OCT. The plasma TMAO levels were measured by using stable isotope dilution liquid chromatography tandem mass spectrometry. Patients were divided into two groups according to the median plasma TMAO level. The prevalence of intimal calcified lesions in the high TMAO group was significantly higher than that in the low TMAO group (90.6 vs. 57.1%, *p* < 0.001; 84.4 vs. 44.4%, *p* < 0.001). After adjustment of traditional risk factors and medication history, patients with calcification in their culprit lesion segments had higher plasma TMAO levels than those without calcification. Moreover, plasma TMAO levels were significantly positively associated with the parameters of calcium burden, including maximal calcification arc (*r* = 0.392, *p* < 0.001), maximal calcification thickness (*r* = 0.443, *p* < 0.001), and calcified length (*r* = 0.466, *p* < 0.001). These results suggested that the level of TMAO is significantly correlated with the incidence of calcification in the culprit lesion segment, and the measurement of TMAO levels might improve clinical management in patients with heavy calcification.

**Clinical Trial Registration:** This study is registered at ClinicalTrials.gov as NCT03593928.

## Introduction

To the best of our knowledge, plaque rupture, plaque erosion, and calcified nodules are three main pathological phenotypes of acute coronary syndromes (ACS) (1). Although calcified nodules are present in <10% of the phenotypes of ACS according to the previous epidemiological analysis, other calcified lesions, including superficial calcified sheets and calcified protrusions, can usually be seen in segments of culprit lesions (2). Identifying the extent and severity of calcification in the segment of the culprit lesion before PCI is crucial because the extent and severity of calcification determine the strategy of management (3). Optical coherence tomography (OCT) is a high-resolution light-based intravascular imaging modality that provides more details to observe vascular calcification and evaluate calcium burden than computed tomography angiography (CTA) or intravascular ultrasound (IVUS) (4), as it allows measurement not only of the length of calcification but also of the arc and thickness (5).

Trimethylamine N-oxide (TMAO), an important gut-microbiota-derived metabolite, has been reported to be a key regulator in the progression of atherosclerosis and plaque instability (6, 7). Moreover, *in vivo* and *vitro* studies demonstrated that plasma TMAO levels are associated with vascular calcification in the aorta (8). However, the association between plasma TMAO levels and calcified lesions in segments of the culprit lesion of the coronary artery in patients with ST-segment–elevation myocardial infarction (STEMI) has not yet been investigated. In this study, we explored the relationship between plasma TMAO concentration and the calcium burden in culprit lesions and their surroundings as determined by OCT in patients with STEMI.

## Methods

### Study Population

**Figure 1:**
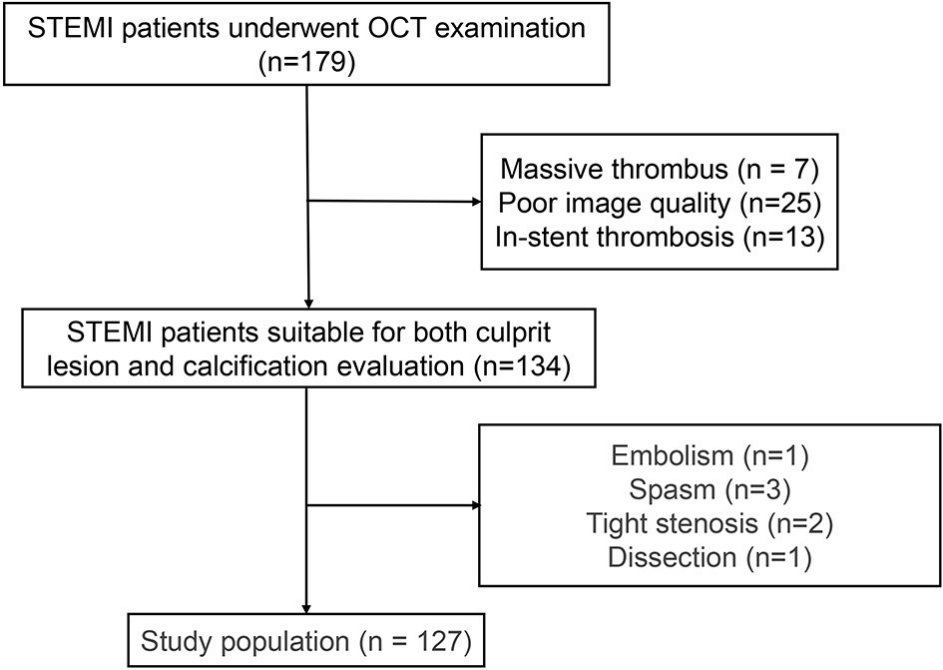
Study flow chart.

From March 2017 to January 2018, a series of 179 eligible patients (age ≥18 years) with STEMI who underwent OCT at Fuwai Hospital were enrolled in our study cohort. The culprit lesions of these patients were evaluated using OCT before interventional procedures. STEMI was defined as continuous chest pain lasting >30 min, ST-segment–elevation >0.1 mV in at least 2 contiguous leads or new left bundle-branch block on the 18-lead ECG, and an elevated troponin I level (9). Patients with cardiac shock, congestive heart failure, and a history of coronary artery bypass graft were excluded. Additionally, those with left main diseases, extremely tortuous vessels, or chronic total occlusion were excluded owing to the difficulty in performing OCT. Of the 179 patients with STEMI who underwent OCT examination, 45 patients were excluded because of massive thrombus (*n* = 7), in-stent thrombosis (*n* = 13) and poor image quality for identifying either culprit lesions or calcifications (*n* = 25). Among the remaining 134 patients who were suitable for both culprit lesion and calcification evaluation, we excluded those with STEMI due to other etiologies, including spasm (*n* = 3), dissection (*n* = 1), tight stenosis (*n* = 2), and embolism (*n* = 1). The study flowchart is displayed in Figure 1. This study was performed in accordance with the Declaration of Helsinki and was approved by the Ethics Committee of Fuwai Hospital. All patients provided written informed consent.

### Acquisition of OCT Images

Patients were administered 300 mg aspirin, 180 mg ticagrelor, or 600 mg clopidogrel and 100 IU/kg heparin before the interventional procedure. Percutaneous coronary intervention was performed via radial or femoral access. Thrombus aspiration was used to reduce the thrombus burden and restore antegrade coronary flow. OCT images of the culprit lesions were acquired with the frequency-domain ILUMIEN OPTIS OCT system and a Dragonfly catheter (St. Jude Medical, Westford, MA) after antegrade blood flow was restored according to the intracoronary imaging technique described previously (10).

### OCT Image Analysis

**Figure 2:**
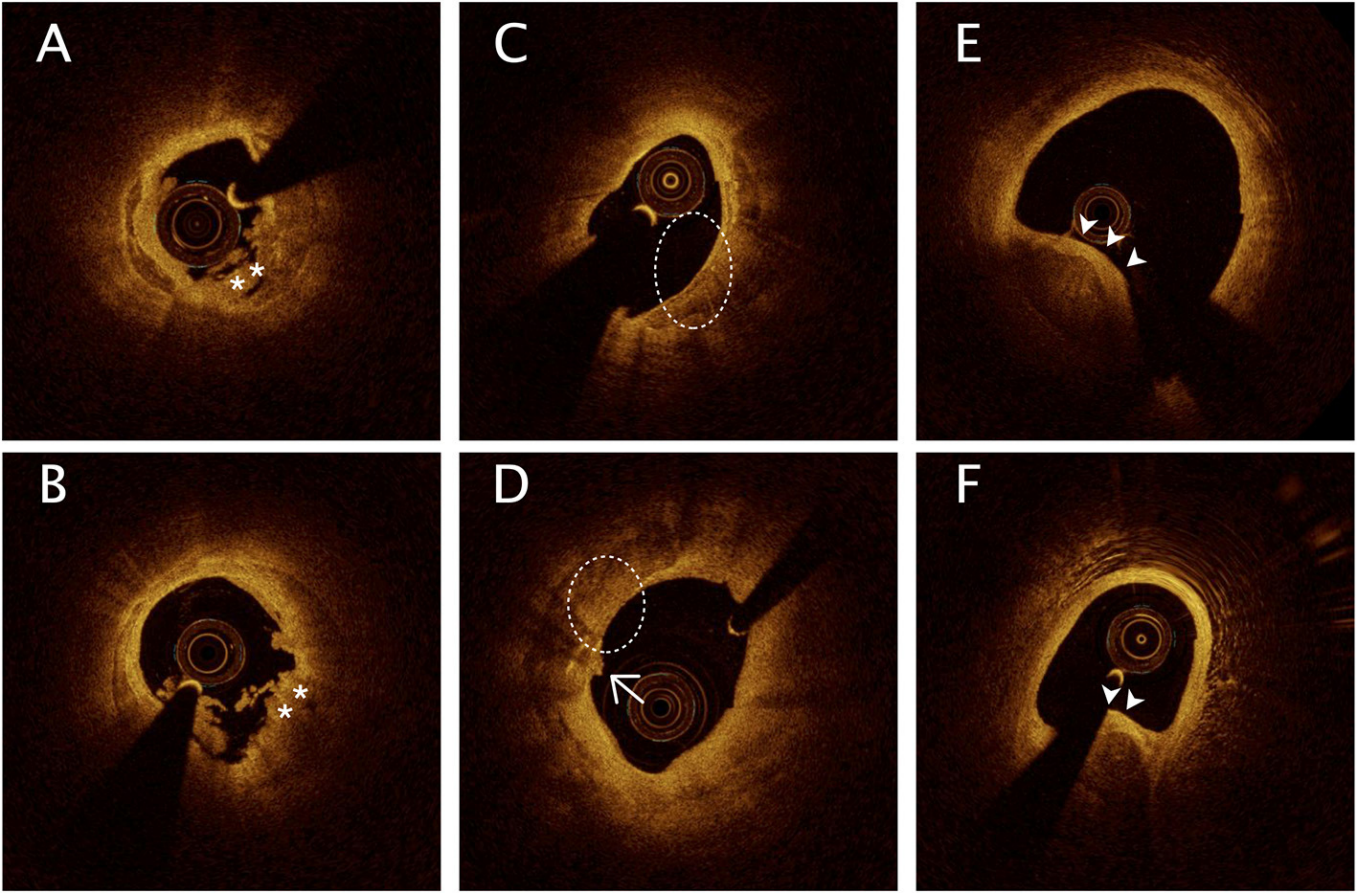
Representative optical coherence tomography images of calcification in culprit lesion segments. **(A,B)** Eruptive calcified nodules with thrombus into the lumen (*). **(C,D)** Superficial calcific sheet (dashed circle) or accompanied by thin fibrous tissue (**D**, arrows). **(E,F)** Calcified protrusion represents protruding calcific into the lumen (arrowheads).

All OCT images were anonymously analyzed on a St. Jude OCT Offline Review Workstation by 3 independent investigators blinded to the other data. Inconsistent results were resolved by discussion among the investigators, who were blinded to the TMAO results. A culprit lesion segment was defined as the entire culprit lesion and its surroundings up to a 3 cm reference segment. According to previously established criteria, plaque rupture (PR) was identified by a disrupted fibrous cap with clear cavity formation, and plaque erosion (PE) was identified by the presence of an attached thrombus overlying an intact and visualized plaque (11). Calcification was identified by the presence of well-delineated low-backscattering heterogeneous regions (11). Calcified lesions were defined by the presence of superficial substantive calcification at or beside the culprit site and were classified into 3 types on the basis of the previous study (2), as shown in Figure 2: (1) eruptive calcified nodules were defined by the expulsion of small calcific nodules into the lumen, (2) superficial calcific sheets were defined by the presence of a sheet-like superficial calcific plate without erupted nodules or protruding mass into the lumen, and (3) calcified protrusion was defined by the presence of a protruding calcific mass without eruptive nodules. The smallest depth of calcium was defined, according to a previous study, as the shortest distance measurable between the leading edge of the calcified plaque and the lumen boundary (12). Quantitative analysis was performed at 1-mm intervals on cross-sectional OCT images. The calcification arc, calcification thickness, and depth of calcium were measured in each cross-sectional image. The calcification length was obtained from the longitudinal view and validated by each cross-sectional image (Supplementary Figure 1).

### Laboratory Tests

Blood samples were collected via radial or femoral access before heparinization using vacutainer tubes containing EDTA. Samples were maintained at 4°C, processed within 3 h, and then stored at −80°C until further analysis. Plasma levels of TMAO were quantified by stable isotope dilution high-performance liquid chromatography with online electrospray ionization tandem mass spectrometry using an API 3200 triple quadrupole mass spectrometer (AB SCIEX, Framingham, MA) with a d9-(trimethyl)-labeled internal standard as described previously (13). The estimated glomerular filtration rate (mL/min per 1.73 m^2^) was calculated using the Modification of Diet in Renal Disease study equation (14).

### Statistical Analysis

Continuous data are presented as the mean ± SD or median (interquartile range). Student's *t*-test or nonparametric test was used for statistical comparisons. Categorical variables are presented as count (percent); comparisons between groups were performed with the χ2 or Fisher exact test. Spearman's correlation analyses were used to examine the correlations between TMAO levels and maximal calcification arc, maximal calcification thickness, calcification length and the smallest depth of calcium. Logistic regression analysis was performed to determine the odds ratio and 95% CI for calcified lesions in the culprit lesion segment stratified according to TMAO as a continuous variable. Adjustments were made for variables including age, sex, smoking, triglycerides, LDL (low-density lipoprotein-cholesterol), HDL (high-density lipoprotein-cholesterol), a history of diabetes mellitus, hypertension, hs-CRP (high-sensitivity C-reactive protein), estimated glomerular filtration rate (eGFR), EF (ejection fraction), and medication history. A 2-tailed *P* < 0.05 was considered statistically significant. The statistical analyses were performed using SPSS software, version 25 (IBM, Armonk, NY).

## Results

### Baseline Characteristics

According to the median TMAO level (2.09 μM), enrolled patients were divided into a high TMAO group (64 patients) and a low TMAO group (63 patients). The baseline characteristics, including age, sex, risk factors, laboratory test results, and medication before hospital, are listed in Table 1. Patients in the high TMAO group tended to be older and present with a history of diabetes mellitus, less smoking and reduced renal function.

**Table 1:** Patient characteristics overall and according to TMAO level.

**Parameters**	**Total (*n* = 127)**	**TMAO≥2.09 μM (*n* = 64)**	**TMAO<2.09 μM (*n* = 63)**	***p* value**
Age	57.9	61.1 ± 10.1	54.7 ± 11.9	0.001
Male, %	101 (79.5)	49 (76.6)	52 (82.5)	0.404
BMI (kg/m^2^)	26.1	25.9 ± 3.0	26.2 ± 3.4	0.580
Hypertension, %	70 (55.1)	33 (51.6)	37 (58.7)	0.417
Diabetes, %	38 (29.9)	25 (39.1)	13 (20.6)	0.023
Hyperlipidemia, %	104 (81.9)	52 (81.3)	52 (82.5)	0.850
Smoking, %	90 (70.9)	40 (62.5)	50 (79.4)	0.036
Stroke, %	14 (11.0)	10 (15.6)	4 (6.5)	0.101
PAD, %	2 (1.6)	2 (3.1)	0 (0)	0.157
Hemoglobin (g/L)	145.6 ± 17.3	142.9 ± 16.4	148.4 ± 17.9	0.076
Platelets (10^9^/L)	230.1 ± 63.7	223.4 ± 63.1	236.9 ± 64.1	0.233
hs-CRP (mg/L)	5.5 (2.3–9.9)	5.3 (2.2–8.5)	5.7 (2.3–11.0)	0.302
eGFR, (mL/min/1.73 m^2^)	97.2 (84.3–108.9)	91.8 (74.9–101.3)	102.6 (91.4–111.6)	<0.001
Triglyceride (mg/dL)	126.6 (78.8–169.2)	126.1 (76.6–169.7)	138.9 (95.6–184.1)	0.471
LDL-C(mg/dL)	114.2 (93.2–135.1)	113.2 (89.6–141.3)	114.6 (93.3–132.7)	0.895
HDL-C (mg/dL)	41.8 (36.0–47.2)	42.0 (34.9–47.1)	41.8 (36.4–47.2)	0.759
EF, %	55.8 ± 6.2	55.9 ± 5.1	55.7 ± 7.2	0.829
Medications before hospital				
Antiplatelet drug	17 (13.4%)	10 (15.6%)	7 (11.1%)	0.455
Statin	19 (15.0%)	12 (18.8%)	7 (11.1%)	0.228
ACEI or ARB	21 (16.5)	11 (17.2)	10 (15.9)	0.842
β-blocker	4 (3.1)	1 (1.6)	3 (4.8)	0.302

*Continuous data are presented as mean ± SD or median (interquartile range), categorical variables are presented as %. BMI indicates body mass index; PAD indicates peripheral artery disease; eGFR, estimated glomerular filtration rate; HDL-C, high-density lipoprotein-cholesterol; hs-CRP, high-sensitivity C-reactive protein; LDL-C, low-density lipoprotein-cholesterol; EF, ejection fraction; ACEI, angiotensin-converting enzyme inhibitor; ARB, angiotensin receptor blocker and TMAO, trimethylamine N-oxide*.

### OCT Findings

OCT findings were compared between the low and high TMAO groups. The incidence of PR in culprit lesions was significantly higher in the high TMAO group than in the low TMAO group (78.1 vs. 19.0%, *p* < 0.001), whereas PE was significantly lower in the high TMAO group (14.1 vs. 76.2%, *p* < 0.001). The prevalence of calcification, especially intimal calcification, in the high TMAO group was significantly higher than that in the low TMAO group (90.6 vs. 57.1%, *p* < 0.001; 84.4 vs. 44.4%, *p* < 0.001) (Supplementary Figure 2). Among intimal calcified lesions, eruptive calcified nodules and superficial calcific sheets were more common in the high TMAO group than in the low TMAO group (17.2 vs. 4.8%, *p* = 0.025; 60.9% vs. 38.1, *p* = 0.01). Calcium burden, including maximal calcification arc, thickness, length and minimum distance to lumen, was greater in the high-level TMAO group than in the low-level TMAO group (Table 2).

**Table 2:** Plaque phenotype and calcification in culprit lesion segments characteristics by optical coherence tomography.

**Parameters**	**Total** **(*n* = 127)**	**TMAO level**		***p* value**
		**High (*n* = 64)**	**Low (*n* = 63)**	
**Culprit plaque types**				
Plaque rupture	62 (48.8)	50 (78.1)	12 (19.0)	<0.001
Plaque erosion	57 (44.9)	9 (14.1)	48 (76.2)	<0.001
Calcified nodules	8 (6.3)	5 (7.8)	3 (4.8)	0.479
Calcification	94 (74.0)	58 (90.6)	36 (57.1)	<0.001
Intimal calcification	82 (64.6)	54 (84.4)	28 (44.4)	<0.001
Medial calcification	15 (11.8)	6 (9.4)	9 (14.3)	0.391
**Intimal calcified lesions**				
Eruptive calcified nodules	14 (11.0)	11 (17.2)	3 (4.8)	0.025
Superficial calcific sheet	63 (49.6)	39 (60.9)	24 (38.1)	0.010
Calcified protrusion	5 (3.9)	4 (6.3)	1 (1.6)	0.177
Maximal calcification arc, degree	90.9 (0–193.1)	146.9 (71.3–227.3)	50.0 (0–228.1)	<0.001
Maximal calcification thickness, μm	530.0 (0–780)	680.0 (500.0–877.5)	290.0 (0–838.0)	<0.001
Calcification length, mm	4.0 (0–11.6)	8.9 (3.5–15.6)	1.6 (0–4.2)	<0.001
Smallest depth of calcium, μm	30.0 (10,0–80.0)	20.0 (10.0–60.0)	50.0 (20.0–107.5)	0.013

*TMAO, Trimethylamine N-oxide*.

The relationship between plasma TMAO level and calcium burden in the culprit lesion segment.

**Figure 3:**
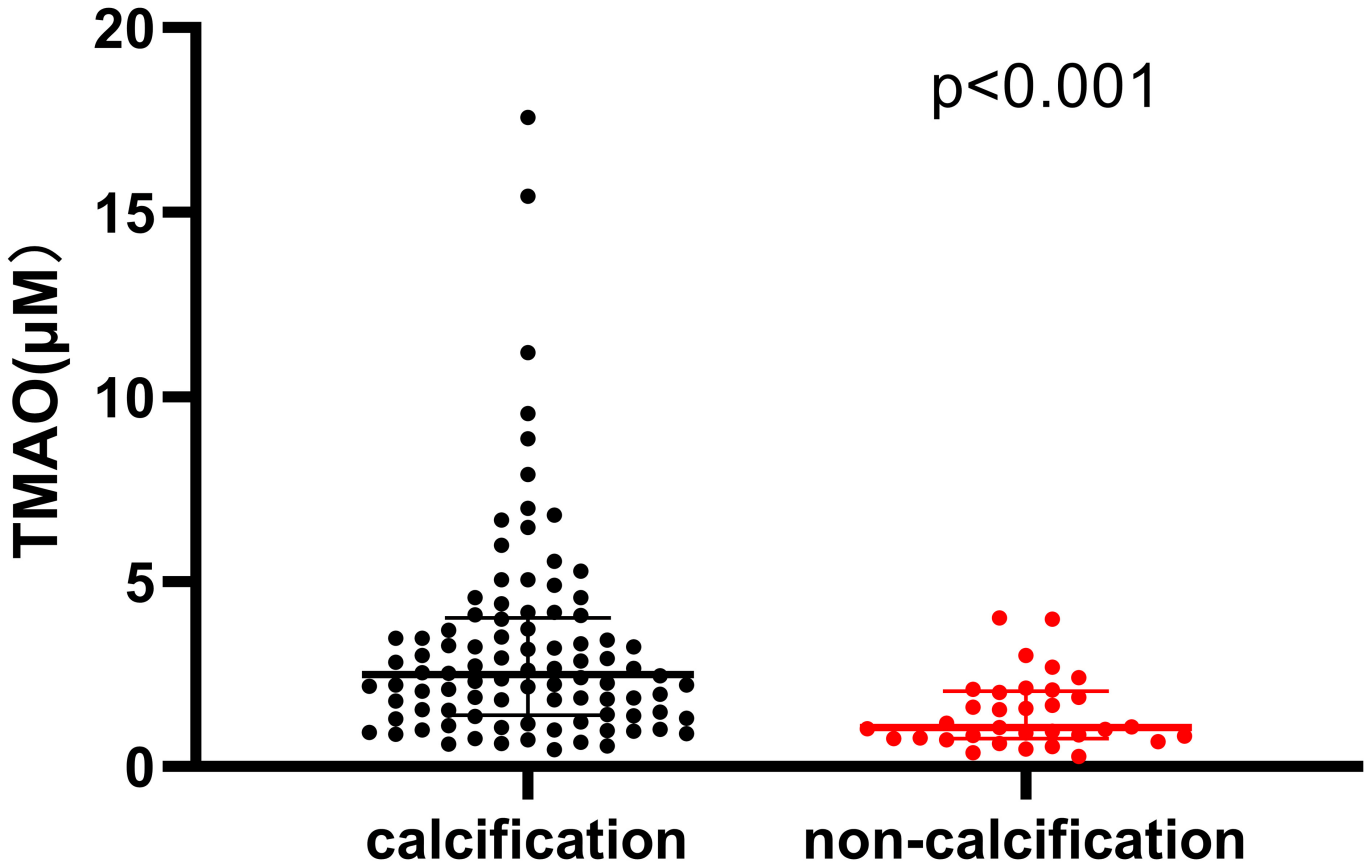
Comparison of plasma trimethylamine N-oxide (TMAO) levels between patients with and without calcification in culprit lesion segment.

**Figure 4:**
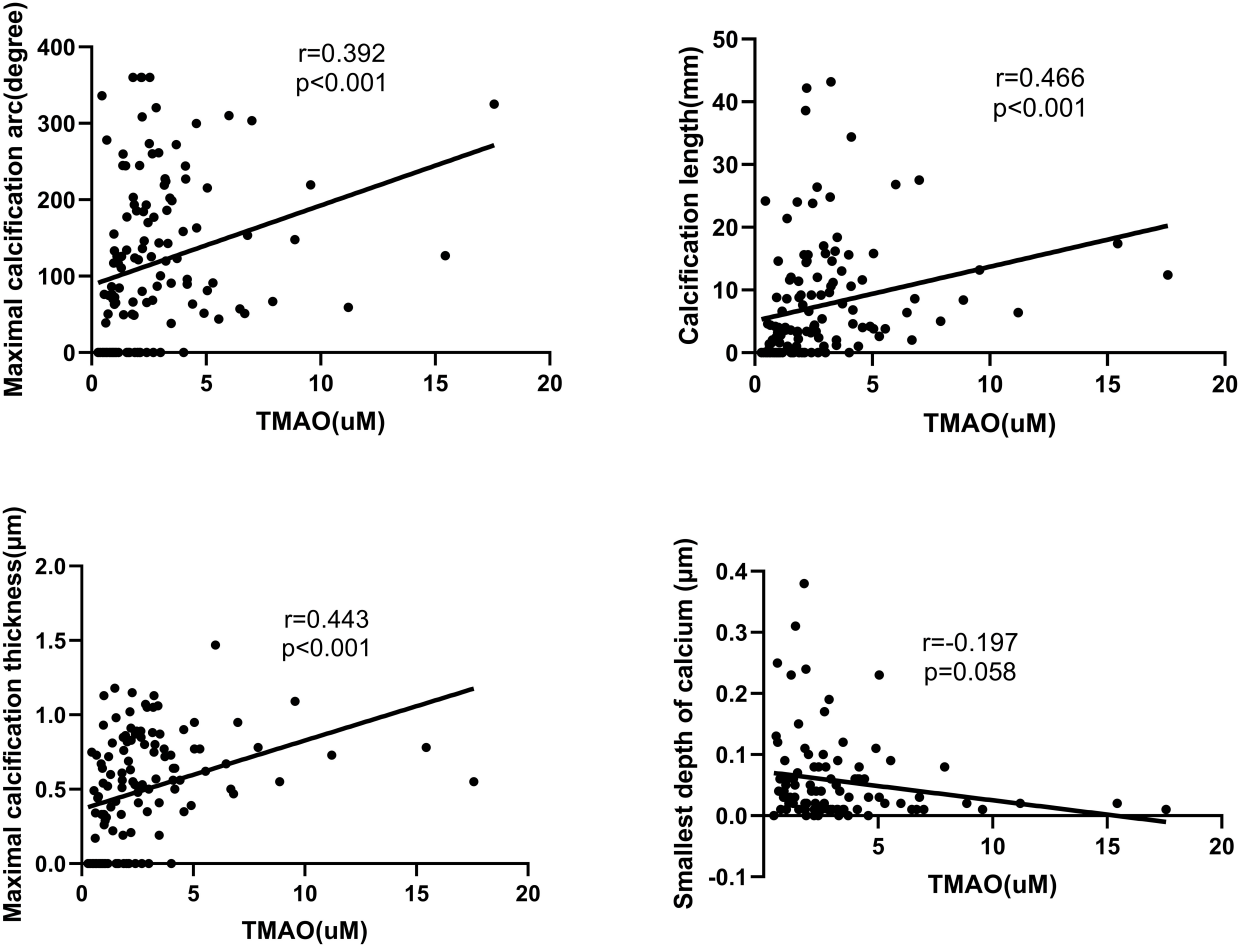
Relations between plasma TMAO levels and parameters of calcification.

Patients with calcification in their culprit lesion segments had higher plasma TMAO levels than those without calcification [2.58 μM (1.45-4.02) vs. 1.40 μM (0.80-2.08), *p* < 0.001] (Figure 3). The multivariate regression analysis showed that age (OR: 1.073, 95% CI: 1.019-1.130, *P* = 0.008) and TMAO (OR: 2.270, 95% CI: 1.358-3.794, *P* = 0.002) were significantly associated with calcification in the culprit lesion segment (Table 3). Additionally, TMAO levels were significantly positively associated with the parameters of calcium burden, including maximal calcification arc (*r* = 0.392, *p* < 0.001), thickness (*r* = 0.443, *p* < 0.001) and length (*r* = 0.466, *p* < 0.001), but were not significantly associated with the smallest depth of calcium (*r* = −0.197, *p* = 0.058) (Figure 4).

**Table 3:** Logistic regression analysis of calcification.

**Variables**	**Univariate**		**Multivariate**	
	**OR (95% CI)**	***p* value**	**OR (95% CI)**	***p* value**
Age	1.060 (1.021–1.100)	0.002	1.073 (1.019–1.130)	0.008
Gender	1.611 (0.554–4.688)	0.382	0.530 (0.076–3.685)	0.521
Hypertension	1.434 (0.647–3.179)	0.374	2.742 (0.881–8.539)	0.082
Diabetes	1.828 (0.715–4.673)	0.208	1.614 (0.505–5.156)	0.419
Smoking	0.574 (0.224–1.471)	0.248	0.870 (0.189–4.014)	0.859
Triglyceride	1.000 (0.996–1.004)	0.984	1.242 (0.778–1.981)	0.364
LDL-C	1.006 (0.994–1.017)	0.345	1.344 (0.675–2.674)	0.400
HDL-C	1.007 (0.963–1.052)	0.762	3.417 (0.366–31.883)	0.281
eGFR	0.983 (0.961–1.005)	0.119	1.008 (0.978–1.040)	0.598
TMAO	2.246 (1.448–3.484)	<0.001	2.270 (1.358–3.794)	0.002
Hs-CRP	0.996 (0.906–1.094)	0.927	1.050 (0.929–1.188)	0.434
EF	0.978 (0.918–1.042)	0.495	0.965 (0.896–1.039)	0.346
Antiplatelet drug	1.164 (0.351–3.856)	0.804	0.931 (0.132–6.583)	0.943
Statin	1.377 (0.422–4.490)	0.596	0.986 (0.113–8.617)	0.990
ACEI/ARB	0.854 (0.301–2.424)	0.767	0.557 (0.089–3.491)	0.532
β-blocker	0.337 (0.046–2.494)	0.287	0.220 (0.017–2.842)	0.246

*Odds ratio shown were for plasma TMAO level as a continuous variable*.

*eGFR, estimated glomerular filtration rate; HDL-C, high-density lipoprotein-cholesterol; hs-CRP, high-sensitivity C-reactive protein; LDL-C, low-density lipoprotein-cholesterol; EF, ejection fraction; ACEI, angiotensin-converting enzyme inhibitor; ARB, angiotensin receptor blocker and TMAO, trimethylamine N-oxide*.

## Discussion

In this study, we found that plasma levels of TMAO were higher in STEMI patients with calcification in their culprit lesion segments than in those without calcification. Moreover, TMAO concentration was significantly positively associated with vascular calcium burden, including maximal calcification arc, maximal calcification thickness and calcification length. Therefore, circulating TMAO levels may serve as a predictor of the severity of calcification in culprit lesion segments in STEMI patients.

Coronary calcification often occurs in advanced atherosclerotic lesions and in regions of apoptotic vascular cells and macrophages (15). High calcium burden is associated with a high incidence of atherosclerotic cardiovascular disease events, including coronary death, nonfatal myocardial infarction, or fatal/nonfatal stroke (16, 17). The underlying mechanism of calcification formation, which is involved in inflammation, advanced atherosclerosis, and metabolic disorders eventually leading to decreased vascular compliance and reduced perfusion (18), has been continuously studied. In ACS patients, calcified lesions are frequently seen in or beside the culprit lesion (19). However, whether these lesions result in plaque instability remains controversial. Early-stage investigation demonstrated that vascular calcified plaques were more stable than noncalcified plaques based on the findings of computed tomography angiography (CTA) (20). However, as the technology of intravascular imaging developed, recent research found that the impact of vessel calcification might be biphasic (21). According to the OCT findings, in culprit lesions of ACS patients, calcification tended to be associated with plaque vulnerability because calcification was more frequently seen in PR patients than in PE patients (69.4 vs. 50%, *p* = 0.074) (22). Additionally, another study revealed similar results (40 vs. 12.8%, *p* = 0.016) (10). In addition, compared with patients with stable angina pectoris, STEMI patients with PR tended to have more calcium deposits (3.5 vs. 2 *p* = 0.08) (23). Moreover, in another study with a cohort of 822 STEMI patients, the prevalence of calcification in patients with PR was significantly higher than that in patients with PE (49.1 vs. 25.8%, *p* < 0.001) (24). However, in nonculprit plaques, patients with greater calcium burden had less vulnerability (19). Collectively, calcification in different amounts, sizes, shapes, and in different regions, may play various roles in plaque homeostasis (25).

Trimethylamine N-oxide (TMAO), an important gut-microbiota-derived metabolite, has been reported to be a key regulator in the progression of atherosclerosis (26). A larger number of studies demonstrated its association with an increased risk of cardiovascular disease in the past few decades (27). Experimental research demonstrated that TMAO could increase the expression of scavenger receptors and inhibit reverse cholesterol transport in macrophages, thus promoting foam cell formation in the arterial wall (6). Additionally, TMAO was proven to be an essential factor of plaque vulnerability, and many studies have provided evidence that TMAO is associated with plaque rupture and vascular inflammation (7, 28). In our study, the rate of plaque rupture in the high TMAO group was also higher than that in the low TMAO group. Although the precise mechanisms of association between TMAO and calcified lesions remain unclear, a recent experimental study provided important clues. They found that TMAO not only promoted osteogenic differentiation of vascular smooth muscle cells *in vitro* but also accelerated vascular calcification *in vivo*, which was mediated by the NLRP3 (nucleotide-binding domain, leucine-rich containing family, pyrin domain-containing-3) inflammasome, and NF-κB (nuclear factor κB) signaling pathways (8). An experimental study also demonstrated that fish-protein-fed mice had elevated levels of TMAO, which were accompanied by an increase in calcification deposits in plaques (29).

However, some clinical studies revealed a weak relationship between plasma TMAO levels and calcification. In young adults, TMAO was not associated with 10-year coronary artery calcium incidence detected by CTA (30). In addition, in HIV-infected subjects, serum levels of trimethylamine (TMA), a precursor of TMAO, were significantly associated with the severity of calcified plaque burden, whereas there was no association of TMAO with coronary plaque features in their cohort (31). In contrast, a recent study revealed that circulating concentrations of TMAO were higher in uremic patients with aortic arch calcification than in those without aortic arch calcification (8). Nevertheless, the results in these studies were merely based on CTA observations and did not evaluate any characteristics of calcified plaques in detail. Recently, in a small-sample study investigating the relationship between TMAO and plaque vulnerability of non-culprit plaque, calcification in the low TMAO group was less than that in the high TMAO group, but not significantly (62.2 vs. 53.3%, *p* = 0.393) (7). To date, the association between plasma TMAO levels and the calcification of culprit lesions in patients with STEMI has not been studied. In our study, the circulating level of TMAO was significantly related to the severity of calcification in the culprit lesion segment, and it may serve as an indicator for evaluating the calcium burden of culprit plaques in clinics.

Although the plasma level of TMAO were significantly positively associated with calcification features including length, thickness, and arc, the correlation of TMAO and these indicators is not strong (0.392 to 0.466). Previous studies revealed that plasma TMAO level could be influenced by many factors such as diet, renal function and even genotype of flavin-containing monooxygenase 3 (32), which may obscure the correlation of TMAO and calcification features. Moreover, the precise mechanism of TMAO and calcification in culprit lesion of STEMI patients remains unclear. Therefore, further investigations into the mechanisms by which elevated TMAO levels contribute to the calcification in culprit lesion and the value of TMAO levels in risk stratification of STEMI patients with calcification are warranted.

Considering that high TMAO deteriorated vascular calcification and plaque stability, it is necessary to maintain it at a low level. Although a vegan diet could decrease the plasma level of TMAO, 3,3-dimethyl-1-butanol (DMB), a structural analog of choline, has been reported to reduce plasma levels of TMAO and atherosclerosis development by inhibiting TMA formation (33). Moreover, animal experiments revealed that antibiotics could efficiently reduce the levels of TMAO and alleviate vascular calcification in CKD rats (8). Additionally, based on these studies, TMAO may be a feasible interventional target in the prevention of vascular calcification and atherosclerosis progression.

This study has some limitations. First, considering the safety and effectiveness, patients with cardiac shock, congestive heart failure, left main diseases, extremely tortuous or heavily calcified vessels, or chronic total occlusion were not enrolled in our study. In addition, patients with a history of coronary artery bypass graft were also excluded. Therefore, selection bias may exist. Second, there are no histopathology data for us to validate OCT findings; thus, some cases might be misinterpreted. For example, it was difficult to differentiate calcific nodules from red thrombi in some patients. Third, due to shallow penetration of OCT, deep calcification cannot be observed in some cases, and the maximum calcification arc and thickness might be underestimated. Forth, although plasma level of TMAO is an independent predictor of calcification in culprit lesion, the correlation of between TMAO levels and calcification features (length, thickness, arc and smallest depth of calcium) are poor. Finally, as the sample size of this study is small, an independent cohort with a large sample size to validate the relationship between TMAO and calcified lesions needs to be established in the future.

## Conclusion

To the best of our knowledge, we demonstrated for the first time an independent relationship between plasma TMAO levels and calcification in culprit lesion segments using OCT in patients with STEMI, which helps to improve the identification and management of calcified lesions in STEMI patients.

## Data Availability Statement

The datasets presented in this article are not readily available because the datasets will be available only by reasonable request. Requests to access the datasets should be directed to hbyanfuwai2018@163.com.

## Ethics Statement

The studies involving human participants were reviewed and approved by the principles of the Declaration of Helsinki and was approved by the review board at Fuwai Hospital. The patients/participants provided their written informed consent to participate in this study.

## Author Contributions

JL and YT analyzed and interpreted the complete data, and were major contributors in writing the manuscript. YW and XZ contributed to the analysis of baseline TMAO concentration. PZ and CL played a leading role in patient enrolment and conducting the registry study. JZ, YC, and RC collected and analyzed the patient data regarding clinical characteristics. HY, LS, and HZ supervised the study and were responsible in funding support. All authors read and approved the final manuscript.

## Conflict of Interest

The authors declare that the research was conducted in the absence of any commercial or financial relationships that could be construed as a potential conflict of interest.
