# Supplementary material for: Association of Trimethylamine N-Oxide Levels and Calcification in Culprit Lesion Segments in Patients With ST-Segment–Elevation Myocardial Infarction Evaluated by Optical Coherence Tomography
Source: Front Cardiovasc Med. 2021 Feb 24;8:628471. doi: 10.3389/fcvm.2021.628471 (PMC7943451; doi:10.3389/fcvm.2021.628471)
Supplement: Supplementary file 1 [file Data_Sheet_1.docx]

**A**  **B**


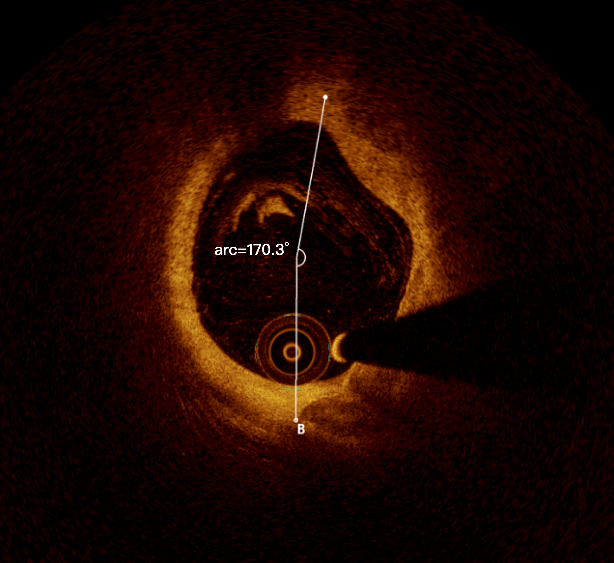

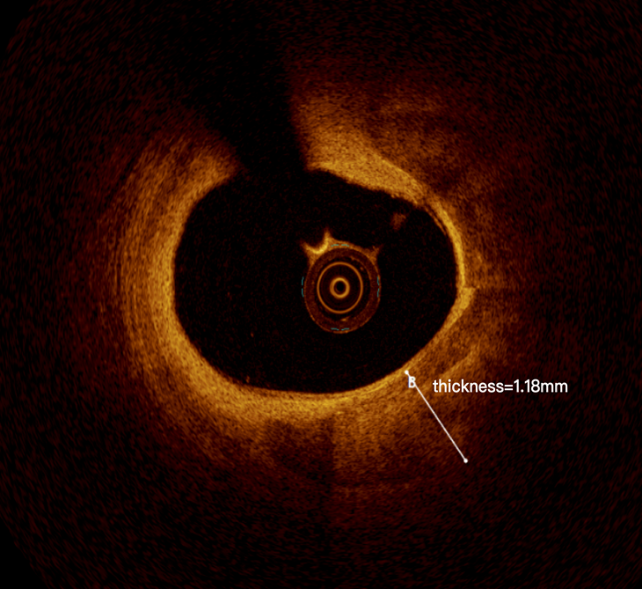


**C**


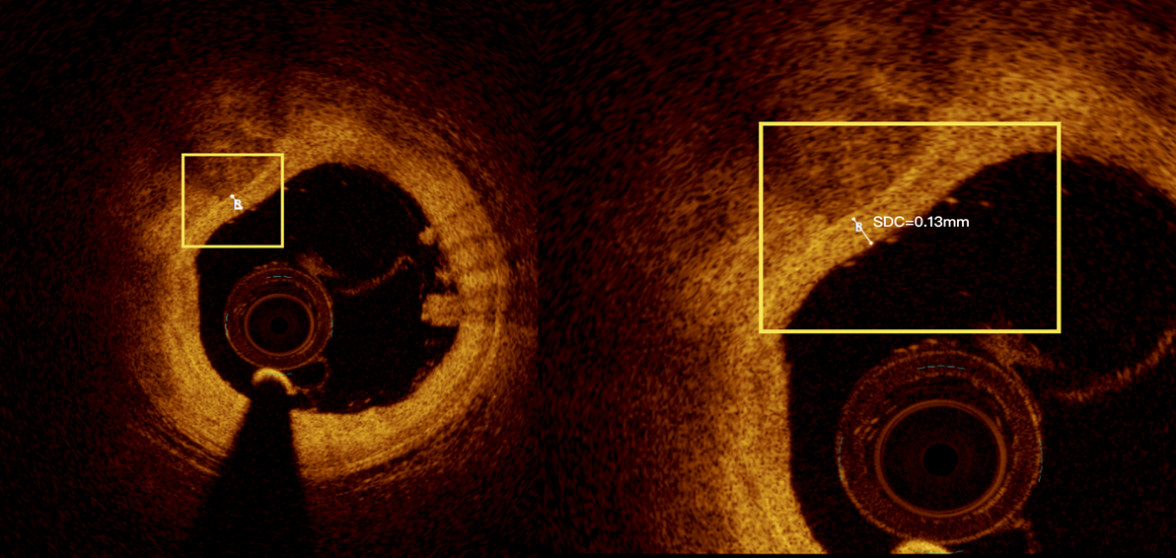


**D**


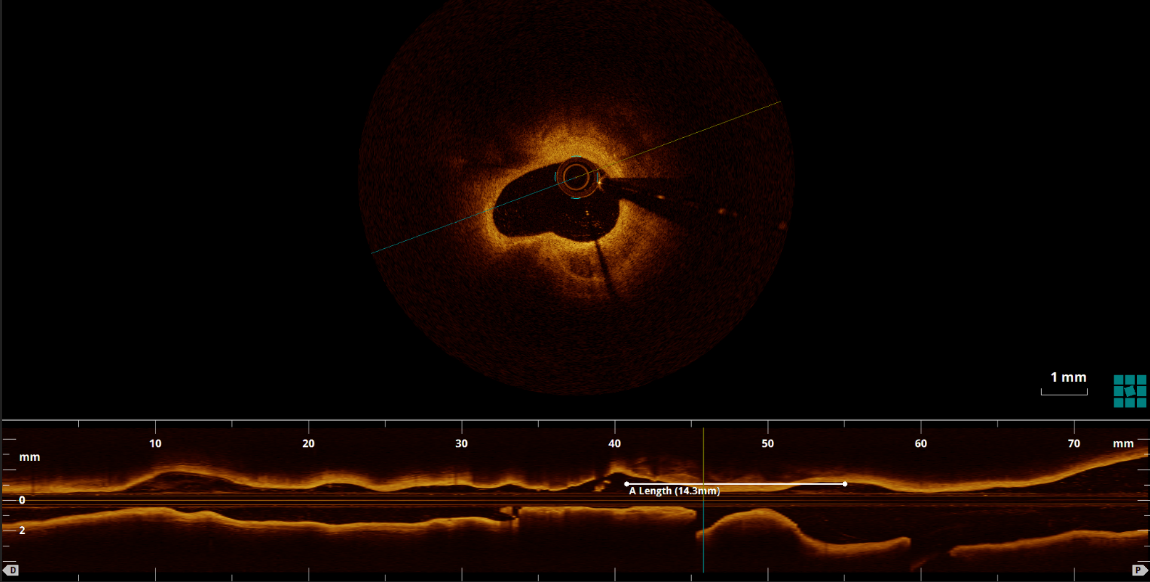


**E**


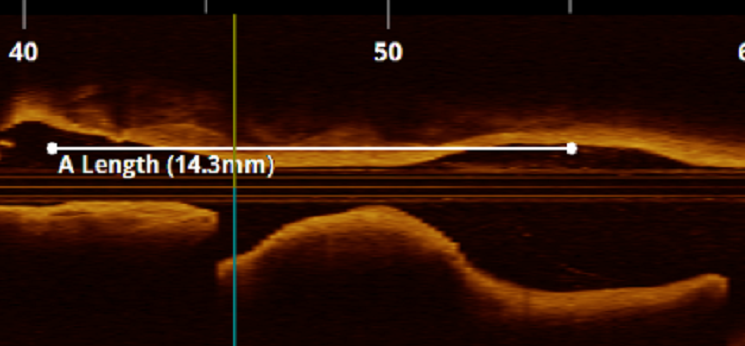


**Figure S1.** Representative optical coherence tomography images for measurement of calcification features.

1. Measurement of maximal arc of calcification. **B.** Measurement of maximal thickness

of calcification. **C.** Measurement of smallest depth of calcium. **D-E.** Measurement of length of calcification from the longitudinal view.

**A B**


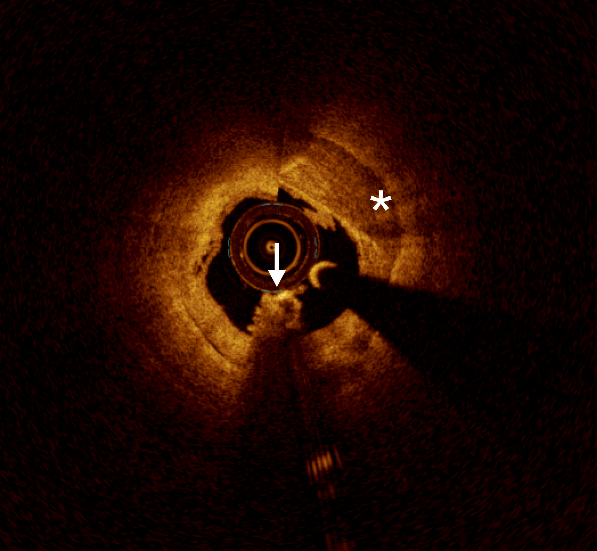

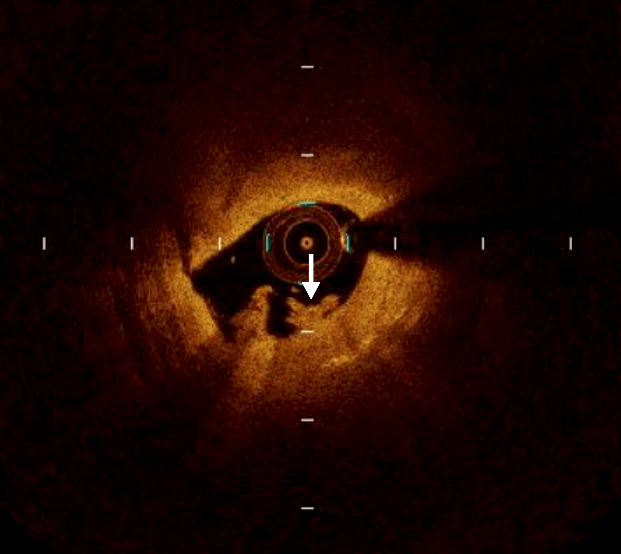


**Figure S2**. Representative optical coherence tomography images of patients with high and low level of TMAO.

1. Patients with high level of TMAO with more calcification (asterisk) and residual white

thrombus (white arrow) in culprit lesion. **B.** Patients with low level of TMAO with no calcification and residual white thrombus (white arrow) in culprit lesion.
